# The association of health literacy with adherence in older adults, and its role in interventions: a systematic meta-review

**DOI:** 10.1186/s12889-015-2251-y

**Published:** 2015-09-17

**Authors:** Bas Geboers, Julii S. Brainard, Yoon K. Loke, Carel J. M. Jansen, Charlotte Salter, Sijmen A. Reijneveld, Andrea F. deWinter

**Affiliations:** Department of Health Sciences, University Medical Center Groningen, University of Groningen, Antonius Deusinglaan 1, FA10, PO Box 196, , 9700 AD Groningen, The Netherlands; Norwich Medical School, Faculty of Medicine & Health Sciences, University of East Anglia, Norwich, UK; Department of Communication and Information Studies, Faculty of Arts, University of Groningen, Groningen, The Netherlands

## Abstract

**Background:**

Low health literacy is a common problem among older adults. It is often suggested to be associated with poor adherence. This suggested association implies a need for effective adherence interventions in low health literate people. However, previous reviews show mixed results on the association between low health literacy and poor adherence. A systematic meta-review of systematic reviews was conducted to study the association between health literacy and adherence in adults above the age of 50. Evidence for the effectiveness of adherence interventions among adults in this age group with low health literacy was also explored.

**Methods:**

Eight electronic databases (MEDLINE, ERIC, EMBASE, PsycINFO, CINAHL, DARE, the Cochrane Library, and Web of Knowledge) were searched using a variety of keywords regarding health literacy and adherence. Additionally, references of identified articles were checked. Systematic reviews were included if they assessed the association between health literacy and adherence or evaluated the effectiveness of interventions to improve adherence in older adults with low health literacy. The AMSTAR tool was used to assess the quality of the included reviews. The selection procedure, data-extraction, and quality assessment were performed by two independent reviewers. Seventeen reviews were selected for inclusion.

**Results:**

Reviews varied widely in quality. Both reviews of high and low quality found only weak or mixed associations between health literacy and adherence among older adults. Reviews report on seven studies that assess the effectiveness of adherence interventions among low health literate older adults. The results suggest that some adherence interventions are effective for this group. The interventions described in the reviews focused mainly on education and on lowering the health literacy demands of adherence instructions. No conclusions could be drawn about which type of intervention could be most beneficial for this population.

**Conclusions:**

Evidence on the association between health literacy and adherence in older adults is relatively weak. Adherence interventions are potentially effective for the vulnerable population of older adults with low levels of health literacy, but the evidence on this topic is limited. Further research is needed on the association between health literacy and general health behavior, and on the effectiveness of interventions.

**Electronic supplementary material:**

The online version of this article (doi:10.1186/s12889-015-2251-y) contains supplementary material, which is available to authorized users.

## Background

Health literacy has been defined as the degree to which people are able to access, understand, appraise, and communicate information to engage with the demands of different health contexts in order to promote and maintain health across the life-course [1]. Health literacy is related to general literacy, but it more specifically encompasses a person’s understanding of health information, both in spoken and in written form [2]. Also, in contrast with general literacy, health literacy is considered a more dynamic and context-dependent ability [3]. Multiple studies have shown that older adults are an especially vulnerable group with regard to health literacy, with rates of low health literacy ranging from 30 to 68 % [4–7], with some studies already finding lower health literacy in adults above the age of 50 [4, 5].

Low health literacy is strongly associated with undesirable health outcomes, such as poor physical fitness [8], higher rates of arthritis and hypertension [9], and higher mortality [10]. It has been suggested that difficulty in adhering to medical advice may partly explain why low health literacy leads to poor health outcomes [11–13]. Adherence can be defined as the extent to which a person’s behavior such as following a diet, taking medication, and/or executing lifestyle changes, are in agreement with recommendations from a health professional [14]. This includes any behavior to prevent, cure, or care for health problems. It also includes many behaviors that are commonly considered to be part of self-management [15]. Rates of poor adherence can be as high as 47 % [16, 17]. Poor adherence has been shown to be associated with several factors, such as poor cognitive abilities [18, 19], a higher number of prescribed medications [18, 19], and the presence of depressive symptoms [20]. Adherence may also be an important factor through which health literacy impacts health outcomes.

Previous reviews have assessed whether an association between health literacy and adherence exists, with mixed results [12, 21, 22]. For example, the reviews of Jin et al. [22] and Witte [12] suggest that high levels of health literacy contribute to successful adherence to therapy. However, Loke et al. [21] found no association between health literacy and adherence in hospitalized patients with diabetes or cardiovascular disease. Also, reviews do not often focus on older adults, which makes it hard to draw conclusions about this specific population. If low health literacy plays an important role in poor adherence among older adults, interventions to improve adherence may be effective to improve the relatively poor health outcomes of older adults with low levels of health literacy. It is, however, unclear whether adherence interventions are effective among older adults with low levels of health literacy.

We conducted a meta-review as a means to adequately assess and summarize a large number of existing reviews and meta-analyses. The meta-review methodology, also called ‘review of reviews’ or ‘overview of reviews’, is a review that only includes systematic reviews and meta-analyses. This methodology is also used by the Cochrane collaboration [23]. It is considered a suitable methodology to summarize existing evidence on topics on which multiple reviews have already been published [23, 24]. Systematic reviews and meta-analyses are considered to be the highest level of evidence. Policy makers and healthcare professionals should make decisions based on systematic reviews, but the vast increase in number of systematic reviews may cause people to become overwhelmed. This is particularly true for topics which are clinically important (e.g. adherence) and a need exists to summarize all findings. Meta-reviews have the potential to identify consistent patterns of results on a large level by taking into account an even larger body of evidence than regular systematic reviews. In a meta-review, differences in the objectives and the quality of the systematic reviews can also be explored. In fact, Smith et al. [25] point out that “A logical and appropriate next step is to conduct a systematic review of reviews of the topic under consideration, allowing the findings of separate reviews to be compared and contrasted, thereby providing clinical decision makers with the evidence they need.” The meta-review methodology has been increasingly used over recent years [26–31].

A broad definition of adherence was adopted for this meta-review, including any behavior that was recommended by health professionals. In modern healthcare, adherence goes beyond medication adherence, and health professionals often advise their patients to perform various other health behaviors, including, for example, doing regular blood glucose checks, increasing physical activity, or decreasing salt intake. Earlier research has also shown that health behaviors are often associated and have shared determinants [32, 33]. This makes it plausible that interventions that aim to improve any kind of adherence may impact on various health behaviors.

In this study, our aims are firstly to evaluate the association between health literacy and adherence in older adults above the age of 50 by performing a meta-review of existing systematic reviews. Secondly, we assess whether interventions to improve adherence are effective among this population.

## Methods

### Search strategy

Systematic searches were conducted for systematic reviews. The searches were conducted in eight electronic databases: MEDLINE, Education Resources Information Center (ERIC), EMBASE, PsycINFO, Cumulative Index to Nursing and Allied Health Literature (CINAHL), DARE, The Cochrane Library, and Web of Knowledge. All databases were searched through September 2014. Combinations of keywords were used, including health literacy, numeracy, adherence, compliance, and self-management. When possible, built-in filters for reviews were used. The full search strategy is presented in Additional file 1. In addition, reference lists of included systematic reviews were manually searched for further reviews that could add to our meta-review.

### Selection of reviews

After the completion of the search and removal of duplicates, two independent reviewers (BG and YKL or JB) screened the titles and abstracts of all articles for potential eligibility for inclusion in our meta-review. Any article selected by at least one of the reviewers was included for full-text review. In the title/abstract review, the inter-rater agreement was around 95 %. Two independent reviewers (BG and JB or YKL) then read the selected articles in full. The reviewers were not blinded to authorship of the reviews. Disagreements in the full-text review were resolved by discussion (BG and JB or YKL).

Reviews were included if they provided information on at least one of our objectives, based on the following criteria:

1. The article was a systematic review (we defined this as a literature review involving a systematic search with application of selection criteria and a description of the number and nature of included studies), either with or without a meta-analysis (i.e. statistical pooling of the results).
2. The review either assessed the association between health literacy and adherence or evaluated the effectiveness of interventions to improve adherence in adults with low health literacy.
3. The review focused on behaviors that need to be maintained for an extended period of time. Reviews that focused on behaviors that are only performed once, such as diagnostic tests and participation in screening, were excluded.
4. At least part of the results of the studies included in the review were specific for the objectives of our meta-review. To confirm this, we verified that the included primary studies considered at least a subset of older adults (mean or median age of at least 50 years) and assessed health literacy with a validated measure, such as the S-TOFHLA [34] or REALM [35]. As an additional criterion, we checked whether the studies were performed in westernized developed countries (USA, Canada, Europe, New Zealand, or Australia).

There were no restrictions regarding type of publication (e.g. report, journal article) or the type of primary studies that were evaluated in the systematic reviews. Also, no restrictions were imposed regarding language. Reviews in non-English languages were translated using online translation services. Native speakers could be contacted in case a non-English review was selected for data-extraction. However, this did not occur.

### Data extraction and quality assessment

Data were extracted from the reviews that met all criteria, using a coding form that captured bibliographic information, the main research question, methodological data, characteristics of the studied population, data about the content and procedures of the included studies, the results, and conclusions as reported by the authors.

The AMSTAR tool was used to check the quality of the included reviews [36]. AMSTAR consists of 11 questions and assesses, among other things, whether a comprehensive literature search is performed, whether duplicate study selection and data-extraction were performed, and whether a full list of included studies is provided. Data-extraction and quality assessment were independently performed by at least two reviewers (BG and JSB or YKL) with disagreements resolved by discussion or by consulting the third reviewer.

### Analyses and reporting

As meta-reviews report on the level of systematic reviews, detailed reporting or pooling of statistics is only possible when at least some of the included systematic reviews conducted a meta-analysis. As we only identified one systematic review with a meta-analysis, a narrative synthesis was used to report our results. First, we summarize the quality of the included reviews. Then, we report on the conclusions of the reviews regarding the association between health literacy and adherence in older adults. Finally, we discuss conclusions of the reviews regarding the effectiveness of adherence interventions in the population of older adults with low levels of health literacy.

In accordance with the meta-review methodology, only information from the systematic reviews is reported in this study. However, to ensure the validity of the results of our meta-review, we also performed data verification by checking whether the reported general conclusions of the reviews were supported by the results of the primary studies that were specifically relevant for our meta-review. A complete overview of these primary studies is presented in Additional file 2.

## Results

### Search results

**Fig. 1:**
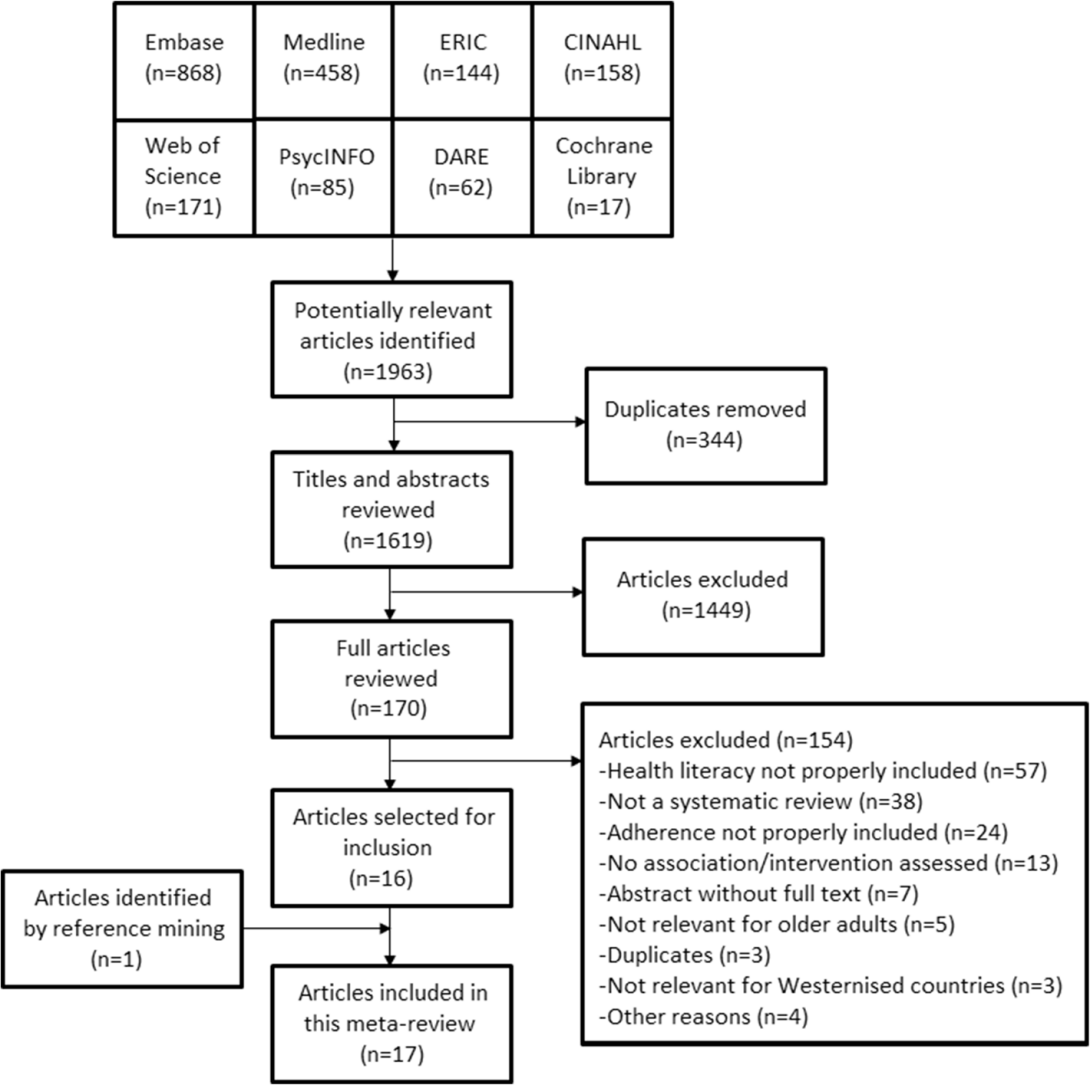
Flowchart of selection procedure

After screening 1619 citations, a total of 17 reviews were included. The full process of selection is presented in Fig. 1.

### Quality assessment

The results of the AMSTAR quality assessment are presented in Table 1. Final AMSTAR quality scores ranged widely from 3 to 10 out of a maximum of 11, with a mean score of 6.4. Nine of the reviews had scores in the range from 7 to 10. Almost all of the reviews conducted a comprehensive search and provided a list and details of the included studies. Ten reviews reported an adequate level of duplicate study selection and data extraction. Only one review statistically pooled the findings of multiple studies [37].

**Table 1:** Results of methodological quality assessment, AMSTAR criteria

Authors	Review on interventions or association	1	2	3	4	5	6	7	8	9	10	11	Total
Al Sayah et al. [46]	Association	+	+	+	+	–	–	+	+	–	–	+	7/11
Berkman et al. [38]	Association	+	+	+	+	–	+	+	+	–	–	+	8/11
Carbone et al. [47]	Interventions	–	–	+	+	+	+	–	–	–	–	+	5/11
Fransen et al. [39]	Both	+	–	–	+	+	+	+	–	–	–	+	6/11
Gellad et al. [40]	Association	+	+	+	+	+	+	–	–	–	+	–	7/11
Keller et al. [41]	Association	+	+	+	+	+	+	+	+	–	–	–	8/11
Lee et al. [48]	Interventions	+	+	+	–	+	+	+	+	–	–	+	8/11
Lewis [42]	Association	+	–	+	–	+	+	–	–	–	–	+	5/11
Loke et al. [21]	Both	+	+	+	–	+	+	+	+	–	+	+	9/11
Newman-Casey et al. [52]	Intervention	+	+	+	–	+	+	+	–	–	+	+	8/11
Ostini and Kairuz [45]	Association	+	–	+	–	+	+	–	–	–	–	+	5/11
Schaefer [51]	Interventions	+	–	–	+	+	+	–	–	–	–	–	4/11
Van Scoyoc et al. [50]	Interventions	+	+	–	+	+	+	–	–	–	–	–	5/11
Sheridan et al. [49]	Interventions	+	+	+	+	+	+	+	+	–	+	+	10/11
Wawrzyniak et al. [44]	Association	+	–	+	–	+	–	–	–	–	–	+	4/11
Weekes [43]	Association	+	–	+	+	–	–	–	–	–	–	–	3/11
Zhang et al. [37]	Association	+	+	+	–	+	+	–	–	+	–	+	7/11

Items in AMSTAR checklist, 1: Pre-specified design, 2: Duplicate screening and data-extraction, 3: Comprehensive literature search, 4: Publication status as criterion, 5: List of selected studies, 6: Characteristics of the studies provided, 7: Validity assessment, 8: Study quality is part of forming conclusions, 9: Valid statistical synthesis of results, 10: Publication bias addressed, 11: Conflict of interest statement

Differences between higher and lower scoring reviews were mostly due to how well they reported on their method of quality assessment and the way of handling the results of quality assessments in formulating conclusions. Eight reviews conducted a quality assessment, six of which adequately used the results of this assessment in formulating their conclusions.

### The association between health literacy and adherence

We included 11 reviews that reported on the association between health literacy and adherence in older adults [21, 37–46]. Two of these reviews also added to our objective regarding interventions on adherence [21, 39]. The conclusions of the selected reviews on the association between health literacy and adherence are presented in Table 2.

**Table 2:** Reviews that examined the association between health literacy and adherence in older adults (*n* = 11)

Authors	Main focus	Total articles (on adherence/specific to this meta-review)	Conclusion on association between health literacy and adherence in older adults
Al Sayah et al. [46]	Improve understanding of relationship between health literacy or numeracy and health outcomes in diabetes.	34 (5/5)	No association with self-care activities.
Berkman et al. [38]	Assess whether low health literacy links to poor health care usage, health outcomes, costs, and disparities in outcomes among all age groups.	111 (17/8)	Evidence of an association was inconsistent.
Fransen et al. [39]	Explore possible associations between health literacy, diabetes self-management, and possible mediators.	11 (11/7^a^)	Very limited evidence for an association with diabetes self-management.
Gellad et al. [40]	Identification of nonfinancial barriers to medication adherence in older adults.	9 (1/1)	No general conclusions about health literacy and adherence are drawn.
Keller et al. [41]	Consider how low health literacy relates to disease state control or medication adherence.	10 (4/3)	Conflicting results about link with adherence.
Lewis [42]	Understand factors associated with adherence to medication in blacks with hypertension.	18 (18/1)	No association was found.
Loke et al. [21]	Review links between health literacy and cardiovascular/diabetes medication adherence.	9 (9/7^a^)	No consistent relationship was found with either condition.
Ostini and Kairuz [45]	Determine what are factors that may influence the possible relationship between health literacy and medication non-adherence.	24 (24^b^/11)	No consistent relationship was found.
Wawrzyniak et al. [44]	Study the current state of knowledge regarding health literacy and health outcomes of HIV-infected individuals.	15 (10/1)	Inconsistent: Some evidence for an association.
Weekes [43]	Overview of health literacy of African American adults.	23 (9/1)	Health literacy influences adherence to medical protocols.
Zhang et al. [37]	Meta-analysis to estimate effect size of the relationship between health literacy and medication adherence.	35 (35/19)	A weak positive association was found.

^a^Including one relevant intervention study

^b^One of these 24 studies is the review of Loke et al. [21] that is also included in this meta-review. The other 23 articles were original research

Six reviews focused on medication adherence specifically. Four of these reviews assessed the association between health literacy and medication adherence, but did not support a strong association [21, 37, 41, 45]. The review of Zhang et al. [37] statistically combined the results of many studies in a meta-analysis. A statistically significant but quite modest association between health literacy and medication adherence was found (*r* < 0.09, *p* < 0.0001 in all analyses). The review of Ostini and Kairuz [45] concluded that addressing medication non-adherence within the framework of health literacy is not as straightforward as was initially assumed, as research often fails to find an association between health literacy and medication adherence. The reviews of Loke et al. [21] and Keller et al. [41] also reported that most of the studies they included did not find significant associations between health literacy and medication adherence. Two other reviews tried to find factors that are associated with medication adherence and considered health literacy as a predictive factor [40, 42]. However, neither concluded that health literacy is an important barrier for medication adherence.

Two reviews focused on the association between health literacy and diabetes in a broad sense. Al Sayah et al. [46] focused on diabetes outcomes and concluded that health literacy is related to diabetes knowledge, but that there is not much evidence for the association between health literacy and other outcomes, including diabetes self-care. Fransen et al. [39] concluded that the evidence for an association between health literacy and various domains of diabetes self-management is very limited.

One review [44] examined the literature on the impact of health literacy on health outcomes in HIV patients, but only one of the included studies focused on older patients. The authors concluded that some studies find an association between health literacy and antiretroviral medication adherence, while other studies fail to find such association.

The review of Berkman et al. [38] focused on the association between health literacy and many different outcomes. The review found evidence on many associations, but concluded that there was insufficient evidence to support an association between health literacy and adherence, despite the inclusion of a relatively large number of studies.

One low quality review concluded that health literacy influences adherence to medical protocols, but the results of the primary studies that are discussed in the review do not support this conclusion [43].

Data verification indicated that the results of the primary studies that are specifically relevant to the current meta-review support the conclusions of the reviews.

### Effectiveness of interventions

Eight reviews provided information on the effects of adherence interventions in older adults with low health literacy [21, 39, 47–52]. An overview of the included reviews is presented in Table 3. No review focused specifically on the effectiveness of interventions on adherence among older adults with low health literacy, and most reviews did not draw general conclusions about this topic. However, the selected reviews discussed the results of one or more intervention studies that provided evidence for our research questions. In order to maximize the amount of information we could extract from the reviews, we also focused on the conclusions that reviews drew about the individual intervention studies, which is a common strategy in meta-reviews [29–31]. In total, the reviews reported on seven different intervention studies. A complete overview of these intervention studies is presented in Additional file 2.

**Table 3:** Reviews that evaluated interventions on adherence in older adults with low health literacy (*n* = 8)

Authors	Main focus	Articles included (relevant to this meta-review)
Carbone et al. [47]	Enhance nutrition advisors’ awareness of health literacy in practice and research.	33 (1)
Fransen et al. [39]^a^	Explore possible associations between health literacy, diabetes self-management, and possible mediators.	11 (1^b^)
Lee et al. [48]	Detect effective strategies to improve health outcomes of low literate patients with cardiovascular disease.	9 (1)
Loke et al. [21]^a^	Review links between health literacy and cardiovascular/diabetes medication adherence.	9 (1)
Newman-Casey et al. [52]	Evaluate educational interventions for glaucoma medication adherence based on quality, efficacy, and extent to which they are grounded in evidence-based Health Behavior Theory.	8 (1)
Schaefer [51]	Find which low health literacy interventions are most effective.	16 (1)
Van Scoyoc et al. [50]	Explore the associations between literacy and diabetes outcomes, and identify clinical strategies likely to be most beneficial.	13 (5)
Sheridan et al. [49]	Identify specific benefits of interventions addressing low health literacy.	39 (1)

^a^Review also provided information about the association between adherence and health literacy. ^b^Another relevant intervention study was described, but not selected, because no results of this intervention were reported

Four reviews on varying topics [39, 47, 49, 51] reported on the same quasi-experimental intervention study [53]. The reviews reported that the study compared various outcomes of diabetes education classes between patients with high and low health literacy, and that low health literacy patients were found to benefit at least as much from diabetes education classes as patients with high health literacy.

In their review, Lee et al. [48] focused on strategies to improve outcomes in low literate patients with cardiovascular disease. One randomized controlled trial is described in which written instructions for medication use were adapted to fit the needs of patients with low health literacy by using a larger font size, decreasing the number of words, and using schema’s with recognizable symbols [54]. The adapted instruction resulted in increased adherence to medication.

In the review by Loke et al. [21] one randomized controlled trial is discussed, in which patients with coronary artery disease and congestive heart failure received instructions for medication use upon being discharged from the hospital [55]. The experimental group received an additional tool that was customized for low literacy patients. After 2 weeks, there was no difference between the two study arms.

In their review on educational interventions to improve glaucoma medication adherence, Newman-Casey et al. [52] identified one randomized controlled trial that focused on health literacy. In this study, glaucoma patients received an educational intervention tailored to their level of health literacy [56]. Trends towards improved adherence were found in the groups with lower reading levels. The review concluded that tailoring information may be useful for educational interventions [52].

Only the review by Von Scoyoc and DeWalt [50], on interventions that aimed to improve diabetes outcomes in patients with low levels of health literacy, reported on more than one intervention that added to our objectives. A three-arm trial showed that both automated telephone disease management and group medical visits led to increased diabetes self-care compared to standard care in people with low literacy [57, 58]. Another study tested an intervention that consisted of a brief counseling session, a low literacy appropriate education guide on diabetes, and follow-up counseling sessions over the telephone [59, 60]. The study found similar improvements in diabetes self-management behavior across patients with different health literacy levels. Finally, staff instructions in the use of a toolkit to facilitate literacy-sensitive diabetes education and management did not lead to improved self-management [61]. The review concluded that it is possible to use interventions to improve the health outcomes of low literate patients with diabetes, but does not draw specific conclusions regarding diabetes self-management [50].

None of the reviews drew any specific conclusions regarding which type of adherence intervention is most suitable for older adults with low health literacy. As a result, data verification was not possible for this question. Also, the small number of described interventions and their large heterogeneity did not allow for strong conclusions.

In their conclusions, many of the reviews stressed the need for further high-quality research in order to strengthen the evidence for interventions among people with low health literacy.

## Discussion

Our meta-review provides only weak evidence in support of an association between health literacy and adherence in older adults. Our results show some evidence that interventions on adherence are effective among older adults with low health literacy, but this evidence is limited.

The results of our systematic meta-review cast doubt on the existence of a strong association between health literacy and adherence among older adults, as the identified systematic reviews only support the existence of a weak association. While non-systematic reviews and health literacy frameworks have suggested that adherence in an important factor through which health literacy impacts health outcomes [11–13], our results do not strongly support this notion.

However, studies on health literacy and adherence in older adults may also have missed a genuine association. First, one of the reviews suggests the possibility of a nonlinear association between health literacy and adherence, in which adherence rates are lowest among those with moderate health literacy [45]. This idea is supported by a survey study that shows that people with low health literacy mostly fail to adhere as a result of a lack of understanding of the given instructions, while people with high health literacy more often non-adhere as a result of deliberately choosing to disregard recommendations [62]. If the association between health literacy and adherence is nonlinear, studies that treat health literacy as a categorical variable with only two or three categories may fail to observe any such associations.

Second, the way in which health literacy and adherence are measured may limit the possibility to draw strong conclusions on the association between the two concepts. The two most commonly used measurement tools for health literacy are the Rapid Estimate of Adult Literacy in Medicine (REALM) and the Short Test of Functional Health Literacy in Adults (S-TOFHLA). Both tools have been criticized for not adequately covering the range of competencies required for adequate health literacy [3, 21]. It has also been suggested that both tools measure limited different elements of health literacy [63]. Also, many tools to measure adherence are based on self-report, but it has been shown that self-reporting is not always an accurate measure of adherence due to people overestimating their adherence, especially when their actual adherence is poor [64].

Third, the inconsistent results on the association between health literacy and adherence may be the result of a confounding effect of age in some studies. Whereas older adults tend to have poorer health literacy [4–7], other research has shown that older age is positively related to adherence [65, 66], which may complicate any association between health literacy and adherence in older adults.

The results of our meta-review on interventions suggest that interventions on adherence are at least as effective for people with low health literacy as for those with high health literacy. The interventions on adherence described in our reviews seem to focus mainly on education and on lowering the health literacy demands of adherence instructions. However, as the reviews provided only limited information on the effectiveness of adherence interventions among older adults with low health literacy, we were not able to draw conclusions regarding which type of intervention could be most beneficial for this population. We identified a clear gap in the available literature, as none of the included systematic reviews drew specific conclusions on the topic of adherence interventions among older adults with low health literacy. Additionally, the input of four of the reviews was based on the results of the same study [53]. In total, the reviews provided information on only seven unique intervention studies.

The reviews included in our meta-review were of varying quality. However, similar conclusions were found among both reviews of higher and lower quality. Our quality assessment indicates that the reviews were mostly based on thorough searches, that the selection procedures and data-extraction were mostly well conducted, and that most reviews gave sufficient information about the included primary studies. However, many of the included reviews did not conduct a quality assessments of the included studies. This is problematic and certainly requires improvement, as it makes it impossible to assess whether conclusions are based on high quality evidence.

### Strengths and limitations

The strengths of our meta-review included the use of a broad definition of adherence, which also includes behaviors outside the cure and care setting, and our extensive search strategy.

However, our meta-review had some limitations. First, we cannot rule out the possibility of selective analysis and outcome reporting in both the primary studies and the reviews. Some research may not have been published if deemed insufficiently novel, positive or significant. Second, as none of our included reviews reported specifically on the effectiveness of adherence interventions among older adults with low health literacy, we could only report on their limited conclusions about individual studies. Third, in many reviews, only part of the studies on the association between health literacy and adherence focused on older adults, leaving unclear to what degree the conclusions of the review are generalizable to this group. However, data verification confirmed that the patterns of results found in the reviews did not change substantially when only considering the primary studies that met the criteria for our meta-review. Finally, in a systematic meta-review, a review based on including systematic reviews, the most recent primary studies may not be covered.

### Implications for public health and future research

Our results suggest that health literacy and adherence exert partially independent effects on the health outcomes of older adults. Public health practitioners should be aware that initiatives that aim to mitigate the negative impacts of low health literacy on health outcomes among older adults should not focus solely on adherence. Initiatives that aim to improve adherence rates among older adults could focus on education and on lowering the health literacy demands of adherence instructions, as evidence on these strategies is the strongest.

Although we adopted a broad definition of adherence in this meta-review, most of the included reviews focused specifically on medication adherence or disease management. None of the reviews focused on adherence with guidelines for general health behavior, such as healthy nutritional behavior and physical activity. Future reviews on the impact of health literacy on the health outcomes of older adults could consider these behaviors as well. Some studies have focused on the association between health literacy and general health behaviors among older adults [67, 68].

Also, despite our extensive search strategy, we found no reviews that focused on improving adherence specifically among older adults with low health literacy. To close this gap in the available literature, future research could focus on reviewing primary studies on specifically this topic, as this could further advance our understanding of the role of health literacy in adherence interventions among older adults. Many of the reviews included in our meta-review stressed the need for more high quality intervention research among people with low health literacy. Further intervention research could indeed help identify which types of interventions are most beneficial for older adults with low health literacy, which could be valuable for clinical practitioners.

## Conclusions

We found inconsistent evidence on the association between health literacy and adherence in older adults in all reviews, including among reviews of higher quality. We are thus unable to support the suggestion that adherence is one of the most important factors through which health literacy impacts health in the vulnerable population of older adults. Nevertheless, our meta-review shows that adherence interventions are potentially beneficial for the vulnerable population of older adults with low levels of health literacy, particularly if they focus on education or lowering the health literacy demands of adherence instructions. However, as the evidence on this topic is limited, our conclusions should be interpreted with caution.

## Additional files

Additional file 1:
**Search strategy of the meta-review.** (DOCX 23 kb) Additional file 2:
**Description of primary studies included in the meta-review.** (DOCX 42 kb)
