# Erratum to: The association of health literacy with adherence in older adults, and its role in interventions: a systematic meta-review

**DOI:** 10.1186/s12889-015-2419-5

**Published:** 2015-10-26

**Authors:** Bas Geboers, Julii S. Brainard, Yoon K. Loke, Carel J. M. Jansen, Charlotte Salter, Sijmen A. Reijneveld, Andrea F. de Winter

**Affiliations:** Department of Health Sciences, University Medical Center Groningen, University of Groningen, Antonius Deusinglaan 1, FA10, Groningen, 9700 AD The Netherlands; Norwich Medical School, Faculty of Medicine & Health Sciences, University of East Anglia, Norwich, UK; Department of Communication and Information Studies, Faculty of Arts, University of Groningen, Groningen, The Netherlands

After the publication of this article it was brought to our attention that the name of the last author, Andrea F. de Winter had been accidentally misspelt as Andrea F. deWinter in the original publication [1]. This has been updated in the article and corrected with this erratum.
