# Supplementary material for: The association of health literacy with adherence in older adults, and its role in interventions: a systematic meta-review
Source: BMC Public Health. 2015 Sep 17;15:903. doi: 10.1186/s12889-015-2251-y (PMC4573285; doi:10.1186/s12889-015-2251-y)
Supplement: Additional file 1: — Search strategy of the meta-review. (DOCX 23 kb) [file 12889_2015_2251_MOESM1_ESM.docx]

**Additional file 1** **Search strategy of the meta-review**

MEDLINE (via EBSCOHost), Date: 15-09-2014

| **No.** | **Strategy** | **Filters** | **Hits** |
| --- | --- | --- | --- |
| S1 | (MH "Health Literacy+") | - | 1,693 |
| S2 | (MH "Patient Medication Knowledge") | - | 53 |
| S3 | (MH "reading" ) | - | 16,998 |
| S4 | "literacy" OR "illiterate" OR "illiteracy" OR "literate" OR "medication knowledge" OR "numeracy" OR "Rapid Estimate of Adult Literacy" OR "Test of Functional Health Literacy" OR "Newest Vital Sign" OR "Short Assessment of Health Literacy" OR "reading" OR "TOFHLA" | - | 126,567 |
| S5 | S1 OR S2 OR S3 OR S4 | - | 126,567 |
| S6 | (MH "Patient Compliance") | - | 46,418 |
| S7 | (MH "Medication Adherence") | - | 7,460 |
| S8 | (MH "Patient Participation") | - | 17,854 |
| S9 | (MH "Guideline Adherence") | - | 20,782 |
| S10 | (MH "Treatment Refusal") | - | 10,530 |
| S11 | (MH "Refusal to Participate") | - | 496 |
| S12 | (MH "Health Behavior") | - | 33,399 |
| S13 | "adherence" OR "patient compliance" OR "patient participation" OR "treatment refusal" OR "nonadherence" OR "non-adherence" OR "noncompliance" OR "non-compliance" OR "self-care" OR "self care" OR "self-management" OR "self management" OR "treatment dropout" OR "treatment drop-out" OR "treatment drop out" OR "pill count" OR "health behavio*" OR "compliance" OR "concordance" | - | 316,860 |
| S14 | S6 OR S7 OR S8 OR S9 OR S10 OR S11 OR S12 OR S13 | - | 317,255 |
| S15 | (MH "Educational Status") | - | 39,539 |
| S16 | “educational status” OR “educational level” OR “educational attainment” | - | 51,615 |
| S17 | S15 OR S16 | - | 51,615 |
| S18 | S5 AND S14 | - | 3,436 |
| S19 | S17 AND S14 | - | 4,480 |
| S20 | S18 OR S19 | Review; Meta-analysis | 458 |

The main search strategy was developed for use in MEDLINE. This search strategy was adapted to suit other databases. Details about the search strategy in other databases can be obtained by contacting the corresponding author.
