# Supplementary material for: The association of health literacy with adherence in older adults, and its role in interventions: a systematic meta-review
Source: BMC Public Health. 2015 Sep 17;15:903. doi: 10.1186/s12889-015-2251-y (PMC4573285; doi:10.1186/s12889-015-2251-y)
Supplement: Additional file 2: — Description of primary studies included in the meta-review. (DOCX 42 kb) [file 12889_2015_2251_MOESM2_ESM.docx]

**Additional file 2 - Description of primary studies included in the meta-review**

| Article | Included in reviews | Type of study | Same sample |
| --- | --- | --- | --- |
| Bains and Egede [1] | Al Sayah et al. [2] Fransen et al. [3] Loke et al. [4] Ostini and Kairuz [5] Zhang et al. [6] | Cross-sectional | Osborn et al. [7] |
| Bauer et al. [8] | Ostini and Kairuz [5] Zhang et al. [6] | Prospective cohort | - |
| Cavanaugh et al. [9] | Fransen et al. [3] | Cross-sectional | Osborn et al. [10] |
| Cavanaugh et al. [11] | Van Scoyoc et al. [12] | RCT | - |
| Cho et al. [13] | Berkman et al. [14] Loke et al. [4] Zhang et al. [6] | Cross-sectional | Gerber et al. [15] |
| Cohen et al. [16] | Zhang et al. [6] | Secondary analysis of RCT | - |
| Cordasco et al. [17] | Loke et al. [4] | RCT | - |
| DeWalt et al. [18] | Van Scoyoc et al. [12] | Pretest posttest intervention | Wallace et al. [19] |
| Estrada et al. [20] | Loke et al. [4] | Prospective cohort | - |
| Fang et al. [21] | Berkman et al. [14] Keller et al. [22] Loke et al. [4] Ostini and Kairuz [5] Zhang et al. [6] | Cross-sectional | - |
| Gatti et al. [23] | Berkman et al. [14] Ostini and Kairuz [5] Weekes [24] Zhang et al. [6] | Cross-sectional | Johnson et al. [25] |
| Gazmararian et al. [26] | Berkman et al. [14] Gellad et al. [27] Keller et al. [22] Loke et al. [4] Ostini and Kairuz [5] Zhang et al. [6] | Prospective cohort | - |
| Gerber et al. [15] | Berkman et al. [14] Loke et al. [4] Zhang et al. [6] | Cross-sectional | Cho et al. [13] |
| Ingram and Ivanov [28] | Zhang et al. [6] | Cross-sectional | - |
| Johnson et al. [25] | Berkman et al. [14] Zhang et al. [6] | Cross-sectional | Gatti et al. [23] |
| Joyner-Grantham et al. [29] | Ostini and Kairuz [5] | Cross-sectional | - |
| Juzych et al. [30] | Zhang et al. [6] | Cross-sectional | - |
| Karter et al. [31] | Fransen et al. [3] | Cross-sectional | - |
| Kim et al. [32] | Al Sayah et al. [2] Carbone et al.[33] Fransen et al.[3] Sheridan et al.[34] Schaefer[35] Zhang et al. [6] | Prospective observational study | - |
| Kripalani et al. [36] | Berkman et al. [14] Loke et al. [4] Ostini and Kairuz [5] Zhang et al. [6] | Cross-sectional | Kripalani et al. [37] |
| Kripalani et al. [37] | Ostini and Kairuz[5] | RCT | Kripalani et al. [36] |
| Lindquist et al. [38] | Zhang et al. [6] | Cross-sectional | - |
| Mancuso [39] | Al Sayah et al. [2] Fransen et al. [3] | Cross-sectional | - |
| Marcum et al. [40] | Zhang et al. [6] | Cross-sectional |  |
| Mbaezue et al. [41] | Al Sayah et al. [2] Fransen et al. [3] | Cross-sectional | - |
| McCleary-Jones [42] | Al Sayah et al. [2] | Cross-sectional | - |
| Mosher [43] | Ostini and Kairuz [5] Zhang et al. [6] | Prospective cohort | - |
| Muir et al. [44] | Keller et al. [22] Zhang et al. [6] | Cross-sectional | - |
| Muir et al. [45] | Newman-Casey et al. [46] | RCT | - |
| Murray et al. [47] | Lee et al. [48] | RCT | Noureldin et al. [49] |
| Nelsen et al. [50] | Wawrzyniak et al. [51] | Cross-sectional | - |
| Noureldin et al.[49] | Ostini and Kairuz [5] Zhang et al. [6] | Secondary analysis of RCT | Murray et al. [47] |
| Osborn et al. [7] | Fransen et al. [3] | Cross-sectional | Bains and Egede [1] |
| Osborn et al. [10] | Ostini and Kairuz [5] Zhang et al. [6] | Cross-sectional | Cavanaugh et al. [9] |
| Schillinger et al. [52] | Van Scoyoc et al. [12] | RCT | Schillinger et al. [53] |
| Schillinger et al. [53] | Van Scoyoc et al. [12] | RCT | Schillinger et al. [52] |
| Schoenthaler et al. [54] | Lewis [55] | Cross-sectional | - |
| Wallace et al. [19] | Van Scoyoc et al. [12] | Pretest posttest intervention | DeWalt et al. [18] |

RCT, Randomized Controlled Trial

**References**

1. Bains SS, Egede LE. Associations between health literacy, diabetes knowledge, self-care behaviors, and glycemic control in a low income population with type 2 diabetes. Diabetes Technol Ther. 2011;13:335-41.

2. Al Sayah F, Majumdar SR, Williams B, Robertson S, Johnson JA. Health literacy and health outcomes in diabetes: a systematic review. J Gen Intern Med. 2013;28:444-52.

3. Fransen MP, von Wagner C, Essink-Bot ML. Diabetes self-management in patients with low health literacy: ordering findings from literature in a health literacy framework. Patient Educ Couns. 2012;88:44-53.

4. Loke YK, Hinz I, Wang X, Salter C. Systematic review of consistency between adherence to cardiovascular or diabetes medication and health literacy in older adults. Ann Pharmacother. 2012;46:863-72.

5. Ostini R, Kairuz T. Investigating the association between health literacy and non-adherence. Int J Clin Pharm. 2014;36:36-44.

6. Zhang NJ, Terry A, McHorney CA. Impact of health literacy on medication adherence: a systematic review and meta-analysis. Ann Pharmacother. 2014;48:741-51.

7. Osborn CY, Bains SS, Egede LE. Health literacy, diabetes self-care, and glycemic control in adults with type 2 diabetes. Diabetes Technol Ther. 2010;12:913-19.

8. Bauer AM, Schillinger D, Parker MM, Katon W, Adler N, Adams AS, Moffet HH, Karter AJ. Health literacy and antidepressant medication adherence among adults with diabetes: the Diabetes Study of Northern California (DISTANCE). J Gen Intern Med. 2013;28:1181-7.

9. Cavanaugh K, Huizinga MM, Wallston KA, Gebretsadik T, Shintani A, Davis D, Gregory RP, Fuchs L, Malone R, Cherrington A, Pignone M, DeWalt DA, Elasy TA, Rothman RL. Association of numeracy and diabetes control. Ann Intern Med. 2008;148:737-46.

10. Osborn CY, Cavanaugh K, Wallston KA, Kripalani S, Elasy TA, Rothman RL, White RO. Health literacy explains racial disparities in diabetes medication adherence. J Health Commun. 2011;16 Suppl 3:268-78.

11. Cavanaugh K, Wallston KA, Gebretsadik T, Shintani A, Huizinga MM, Davis D, Gregory RP, Malone R, Pignone M, DeWalt D, Elasy TA, Rothman RL. Addressing literacy and numeracy to improve diabetes care: two randomized controlled trials. Diabetes Care. 2009;32:2149-55.

12. Van Scoyoc EE, DeWalt DA. Interventions to improve diabetes outcomes for people with low literacy and numeracy: a systematic literature review. Diabetes Spectrum. 2010;23:228-37.

13. Cho YI, Lee SD, Arozullah AM, Crittenden KS. Effects of health literacy on health status and health service utilization amongst the elderly. Soc Sci Med. 2008;66:1809-16.

14. Berkman ND, Sheridan SL, Donahue KE, Halpern DJ, Crotty K. Low health literacy and health outcomes: an updated systematic review. Ann Intern Med. 2011;155:97-107.

15. Gerber BS, Cho YI, Arozullah AM, Lee SD. Racial differences in medication adherence: a cross-sectional study of Medicare enrollees. Am J Geriatr Pharmacother. 2010;8:136-45.

16. Cohen MJ, Shaykevich S, Cawthon C, Kripalani S, Paasche-Orlow M, Schnipper JL. Predictors of medication adherence postdischarge: the impact of patient age, insurance status, and prior adherence. J Hosp Med. 2012;7:470-5.

17. Cordasco KM, Asch SM, Bell DS, Guterman JJ, Gross-Schulman S, Ramer L, Elkayam U, Franco I, Leatherwood CL, Mangione CM. A low-literacy medication education tool for safety-net hospital patients. Am J Prev Med. 2009;37:S209-S216.

18. DeWalt DA, Davis TC, Wallace AS, Seligman HK, Bryant-Shilliday B, Arnold CL, Freburger J, Schillinger D. Goal setting in diabetes self-management: taking the baby steps to success. Patient Educ Couns. 2009;77:218-23.

19. Wallace AS, Seligman HK, Davis TC, Schillinger D, Arnold CL, Bryant-Shilliday B, Freburger JK, DeWalt DA. Literacy-appropriate educational materials and brief counseling improve diabetes self-management. Patient Educ Couns. 2009;75:328-33.

20. Estrada CA, Martin-Hryniewicz M, Peek BT, Collins C, Byrd JC. Literacy and numeracy skills and anticoagulation control. Am J Med Sci. 2004;328:88-93.

21. Fang MC, Machtinger EL, Wang F, Schillinger D. Health literacy and anticoagulation-related outcomes among patients taking warfarin. J Gen Intern Med. 2006;21:841-6.

22. Keller DL, Wright J, Pace HA. Impact of health literacy on health outcomes in ambulatory care patients: a systematic review. Ann Pharmacother. 2008;42:1272-81.

23. Gatti ME, Jacobson KL, Gazmararian JA, Schmotzer B, Kripalani S. Relationships between beliefs about medications and adherence. Am J Health Syst Pharm. 2009;66:657-64.

24. Weekes CV. African Americans and health literacy: a systematic review. ABNF J. 2012;23:76-80.

25. Johnson VR, Jacobson KL, Gazmararian JA, Blake SC. Does social support help limited-literacy patients with medication adherence? A mixed methods study of patients in the Pharmacy Intervention for Limited Literacy (PILL) study. Patient Educ Couns. 2010;79:14-24.

26. Gazmararian JA, Kripalani S, Miller MJ, Echt KV, Ren J, Rask K. Factors associated with medication refill adherence in cardiovascular-related diseases: a focus on health literacy. J Gen Intern Med. 2006;21:1215-21.

27. Gellad WF, Grenard JL, Marcum ZA. A systematic review of barriers to medication adherence in the elderly: looking beyond cost and regimen complexity. Am J Geriatr Pharmacother. 2011;9:11-23.

28. Ingram RR, Ivanov LL. Examining the association of health literacy and health behaviors in African American older adults: does health literacy affect adherence to antihypertensive regimens? J Gerontol Nurs. 2013;39:22-32.

29. Joyner-Grantham J, Mount DL, McCorkle OD, Simmons DR, Ferrario CM, Cline DM. Self-reported influences of hopelessness, health literacy, lifestyle action, and patient inertia on blood pressure control in a hypertensive emergency department population. Am J Med Sci. 2009;338:368-72.

30. Juzych MS, Randhawa S, Shukairy A, Kaushal P, Gupta A, Shalauta N. Functional health literacy in patients with glaucoma in urban settings. Arch Ophthalmol. 2008;126:718-24.

31. Karter AJ, Subramanian U, Saha C, Crosson JC, Parker MM, Swain BE, Moffet HH, Marrero DG. Barriers to insulin initiation: the translating research into action for diabetes insulin starts project. Diabetes Care. 2010;33:733-5.

32. Kim S, Love F, Quistberg DA, Shea JA. Association of health literacy with self-management behavior in patients with diabetes. Diabetes Care. 2004;27:2980-2.

33. Carbone ET, Zoellner JM. Nutrition and health literacy: a systematic review to inform nutrition research and practice. J Acad Nutr Diet. 2012;112:254-65.

34. Sheridan SL, Halpern DJ, Viera AJ, Berkman ND, Donahue KE, Crotty K. Interventions for individuals with low health literacy: a systematic review. J Health Commun. 2011;16 Suppl 3:30-54.

35. Schaefer CT. Integrated review of health literacy interventions. Orthop Nurs. 2008;27:302-17.

36. Kripalani S, Gatti ME, Jacobson TA. Association of age, health literacy, and medication management strategies with cardiovascular medication adherence. Patient Educ Couns. 2010;81:177-81.

37. Kripalani S, Schmotzer B, Jacobson TA. Improving Medication Adherence through Graphically Enhanced Interventions in Coronary Heart Disease (IMAGE-CHD): a randomized controlled trial. J Gen Intern Med. 2012;27:1609-17.

38. Lindquist LA, Go L, Fleisher J, Jain N, Friesema E, Baker DW. Relationship of health literacy to intentional and unintentional non-adherence of hospital discharge medications. J Gen Intern Med. 2012;27:173-78.

39. Mancuso JM. Impact of health literacy and patient trust on glycemic control in an urban USA population. Nurs Health Sci. 2010;12:94-104.

40. Marcum ZA, Zheng Y, Perera S, Strotmeyer E, Newman AB, Simonsick EM, Shorr RI, Bauer DC, Donohue JM, Hanlon JT. Prevalence and correlates of self-reported medication non-adherence among older adults with coronary heart disease, diabetes mellitus, and/or hypertension. Res Social Adm Pharm. 2013;9:817-27.

41. Mbaezue N, Mayberry R, Gazmararian J, Quarshie A, Ivonye C, Heisler M. The impact of health literacy on self-monitoring of blood glucose in patients with diabetes receiving care in an inner-city hospital. J Natl Med Assoc. 2010;102:5-9.

42. McCleary-Jones V. Health literacy and its association with diabetes knowledge, self-efficacy and disease self-management among African Americans with diabetes mellitus. ABNF J. 2011;22:25-32.

43. Mosher HJ, Lund BC, Kripalani S, Kaboli PJ. Association of health literacy with medication knowledge, adherence, and adverse drug events among elderly veterans. J Health Commun. 2012;17 suppl 3:241-51.

44. Muir KW, Santiago-Turla C, Stinnett SS, Herndon LW, Allingham RR, Challa P, Lee PP. Health literacy and adherence to glaucoma therapy. Am J Ophthalmol. 2006;142:223-6.

45. Muir KW, Ventura A, Stinnett SS, Enfiedjian A, Allingham RR, Lee PP. The influence of health literacy level on an educational intervention to improve glaucoma medication adherence. Patient Educ Couns. 2012;87:160-64.

46. Newman-Casey PA, Weizer JS, Heisler M, Lee PP, Stein JD. Systematic review of educational interventions to improve glaucoma medication adherence. Semin Ophthalmol. 2013;28:191-201.

47. Murray MD, Young J, Hoke S, Tu W, Weiner M, Morrow D, Stroupe KT, Wu J, Clark D, Smith F, Gradus-Pizlo I, Weinberger M, Brater DC. Pharmacist intervention to improve medication adherence in heart failure: a randomized trial. Ann Intern Med. 2007;146:714-25.

48. Lee TW, Lee SH, Kim HH, Kang SJ. Effective intervention strategies to improve health outcomes for cardiovascular disease patients with low health literacy skills: A systematic review. Asian Nurs Res. 2012;6:128-36.

49. Noureldin M, Plake KS, Morrow DG, Tu W, Wu J, Murray MD. Effect of health literacy on drug adherence in patients with heart failure. Pharmacotherapy. 2012;32:819-26.

50. Nelsen A, Gupta S, Trautner BW, Petersen NJ, Garza A, Giordano TP, Naik AD, Rodriguez-Barradas M. Intention to adhere to HIV treatment: a patient-centred predictor of antiretroviral adherence. HIV Med. 2013;14:472-80.

51. Wawrzyniak AJ, Ownby RL, McCoy K, Waldrop-Valverde D. Health literacy: impact on the health of HIV-infected individuals. Curr HIV/AIDS Rep. 2013;10:295-304.

52. Schillinger D, Hammer H, Wang F, Palacios J, McLean I, Tang A, Youmans S, Handley M. Seeing in 3-D: examining the reach of diabetes self-management support strategies in a public health care system. Health Educ Behav. 2008;35:664-82.

53. Schillinger D, Handley M, Wang F, Hammer H. Effects of self-management support on structure, process, and outcomes among vulnerable patients with diabetes: a three-arm practical clinical trial. Diabetes Care. 2009;32:559-66.

54. Schoenthaler A, Chaplin WF, Allegrante JP, Fernandez S, Diaz-Gloster M, Tobin JN, Ogedegbe G. Provider communication effects medication adherence in hypertensive African Americans. Patient Educ Couns. 2009;75:185-91.

55. Lewis L. Factors associated with medication adherence in hypertensive blacks: a review of the literature. J Cardiovasc Nurs. 2012;27:208-19.
